# Arabian Method of Treating Stone in the Bladder

**Published:** 1852-09

**Authors:** 


ART. VI — *Arabian Method of treating Stone in the Bladder.*

AUGUST 10th, 1852.

In an old work, published in London in the year 1743, entitled A View of the Levant, &c., by Charles Perry, M. D., there is described a curious method of treating a very formidable disease, which is practiced by the Arabs in the city of Cairo. Thinking it might interest your readers, as a medical curiosity, I have copied it out word for word, merely modernising the spelling a little. Yours truly, G. H.

" Being the other day in discourse with one Signior Gabrieli, a Venetian who has practiced here as a Medico for many years, he entertained us with an account of a great cure which he had lately performed. His story was thus, or to this purpose : That a certain Effendi, a person of great affairs and consideration, and about fifty years old, had been tormented with violent and continued pains in his reins, for twelve years past, without intermission: that during this long and irksome time of twelve years misery, he had applied himself to all the doctors, (whether real or nominal,) that he could meet with or hear of in this city, but without any sort of benefit; for they all alike mistook his case, judging it to be no other than a cold, which had determined and fixed itself upon that region. At length, about eight months ago, good luck or providence directed him to this Signior Gabrieli. He was no sooner called, and fully instructed of the patient's complaint, than he judged and pronounced it to be of the nephritic kind; but he judged much better of the disease than of the medicines he applied for the cure, for he gave nothing but mallow water in large quantity, with oils and syrups to lubricate. These indeed were very innocent remedies, and, as one would be apt to think, equally impotent, as in fact they proved. But Signior Gabrieli, having experienced those, and such like things, for a considerable time, without any fruit or effect, and being acquainted with an Arab, who was famed for his dexterity in blowing wind up the penis for the cure of stone and gravel, he went in search of him, carried him to the patient, and ordered him to perform the operation without delay, in the best manner he could. The operator, having his instruments about him, went to work directly. He first ordered the patient to stand up in an erect posture; then he put the end of a common cane pipe, (which was about three inches long, and cut taper, after the manner of our penis syringes,) into the urethra; and the instrument being adjusted in such a manner as he thought proper, he blew into it, with all his might, for a considerable time; then, holding the urethra, to prevent the wind's flying out again, he played about the bottom of his belly with the other hand, (especially above the os pubis and near the groins,) for a considerable time; then, relieving the urethra, he let the wind discharge itself, beating his belly gently with his hand, to force the wind out with a greater impetus. When the wind was pretty well discharged, he applied a pipe again into the urethra, and then sucked with the same force as he had before blown. By this first operation the patient voided eleven stones near as big as vetches ; and the same operation being repeated every morning and evening, till he was entirely freed from pain, and from all further emissions of stone or gravel, the whole quantity of stones discharged, (besides an incredible quantity of gravel,) amounted to near three tea cups full; and besides these, he excreted a great deal of white viscous matter in his urine.

"However, we confess, we were rather pleased and diverted with this story, than satisfied about it; because people are generally partial in their own favor; or at least will exaggerate in their accounts of things which tend to their own glory and honor. We therefore desired Signior Gabrieli, for our full satisfaction and conviction, to carry us to see the person. Signior Gabrieli replied, without the least scruple or hesitation, that we were masters to go whenever we would; and no longer ago than yesterday, we went and had an interview with this Effendi. We saw the stones which he had voided, and had all the other circumstances of the cure confirmed by the patient's own mouth. Most of the stones are as big as vetches, and somewhat of the same figure; they are all of a dark yellow color, and of a friable texture. The Effendi told us that he had not been able to mount his horse, nor scarce to move about the house, for the space of twelve years before, but was now pretty well, and very easy. He said, however, that when he urined, he had yet a burning pain in the urethra, near its extremity; and examining his urine, we found it of a wheyish color, abounding with a number of white filaments."

